# IFN-γ independent markers of *Mycobacterium tuberculosis* exposure among male South African gold miners

**DOI:** 10.1016/j.ebiom.2023.104678

**Published:** 2023-06-26

**Authors:** Leela R.L. Davies, Malisa T. Smith, Deniz Cizmeci, Stephanie Fischinger, Jessica Shih-Lu Lee, Lenette L. Lu, Erik D. Layton, Alison D. Grant, Katherine Fielding, Catherine M. Stein, W. Henry Boom, Thomas R. Hawn, Sarah M. Fortune, Robert S. Wallis, Gavin J. Churchyard, Galit Alter, Chetan Seshadri

**Affiliations:** a Ragon Institute of MGH, MIT, and Harvard, Cambridge, MA, USA; b Brigham and Women's Hospital, Boston, MA, USA; c Department of Medicine, University of Washington School of Medicine, Seattle, WA, USA; d Department of Biological Engineering, Massachusetts Institute of Technology, Cambridge, MA, USA; e TB Centre, London School of Hygiene and Tropical Medicine, London, UK; f Department of Medicine, Case Western Reserve University, Cleveland, OH, USA; g Department of Population & Quantitative Health Sciences, Case Western Reserve University, Cleveland, OH, USA; h Department of Immunology and Infectious Diseases, Harvard TH Chan School of Public Health, Boston, MA, USA; i The Aurum Institute, Parktown, South Africa; j Department of Medicine, Vanderbilt University, Nashville, TN, USA; k Moderna Therapeutics, Cambridge, MA, USA; l Seattle Tuberculosis Research Advancement Center, Seattle, WA, USA

**Keywords:** Human, T cell, B cell, Antibody, *Mycobacterium tuberculosis*, Resister, Immunology

## Abstract

**Background:**

The prevalence of tuberculosis among men who work in the gold mines of South Africa is among the highest in the world, but a fraction of miners demonstrate consistently negative results upon tuberculin skin test (TST) and IFN-γ release assay (IGRA). We hypothesized that these “resisters” (RSTRs) may display unconventional immune signatures of exposure to *M. tuberculosis* (M.tb).

**Methods:**

In a cohort of RSTRs and matched controls with latent TB infection (LTBI), we profiled the functional breadth of M.tb antigen-specific T cell and antibody responses using multi-parameter flow cytometry and systems serology, respectively.

**Findings:**

RSTRs and LTBI controls both exhibited IFN-γ independent T-cell and IgG antibody responses to M.tb-specific antigens ESAT-6 and CFP-10. Antigen-specific antibody Fc galactosylation and sialylation were higher among RSTRs. In a combined T-cell and antibody analysis, M.tb lysate-stimulated TNF secretion by T cells correlated positively with levels of purified protein derivative-specific IgG. A multivariate model of the combined data was able to differentiate RSTR and LTBI subjects.

**Interpretation:**

IFN-γ independent immune signatures of exposure to M.tb, which are not detected by approved clinical diagnostics, are readily detectable in an occupational cohort uniquely characterized by intense and long-term infection pressure. Further, TNF may mediate a coordinated response between M.tb-specific T-cells and B-cells.

**Funding:**

This work was supported by the 10.13039/100000002US National Institutes of Health (R01-AI124348 to Boom, Stein, and Hawn; R01-AI125189 and R01-AI146072 to Seshadri; and 75N93019C00071 to Fortune, Alter, Seshadri, and Boom), the 10.13039/100000862Doris Duke Charitable Foundation (Davies), the 10.13039/100000865Bill & Melinda Gates Foundation (OPP1151836 and OPP1109001 to Hawn; and OPP1151840 to Alter), Mass Life Science Foundation (Fortune), and Good Ventures Fund (Fortune).

**Research in context**

**Evidence before this study**

A small fraction of individuals that are highly exposed to *Mycobacterium tuberculosis* (M.tb) never test positive for infection or clinical disease. In a prior study of Ugandan household contacts, we showed that these “resisters” exhibit M.tb-specific IFN-γ independent T cell and antibody responses consistent with their epidemiologic history. The potential protective immunologic mechanisms that “resisters” might harbor remain undefined.

**Added value of this study**

While prior studies of “resisters” have mostly focused on household or community exposure, here we examined a unique high-burden, long-term, occupationally exposed population of male South African gold miners. In a completely independent setting, we again demonstrated that “resisters” harbor IFN-γ independent M.tb specific T and B cell responses. In addition, we newly identified modifications in M.tb-specific antibody Fc glycosylation, including increased galactosylation and sialylation, that were uniquely detected among “resisters.” Finally, we identified TNF as a potential mediator for M.tb-specific T and B cell interactions among these individuals.

**Implications of all the available evidence**

This work adds to the mounting evidence that, despite having negative clinical testing for M.tb, “resisters” show clear evidence of adaptive immune priming in response to M.tb exposure. Immune phenotypes identified in this and similar cohorts may inform our understanding of protective immunity to M.tb infection, leading the way to the development of new therapeutic or vaccine strategies.

## Introduction

*Mycobacterium tuberculosis* (M.tb) is a leading infectious cause of death, responsible for 1.4 million deaths worldwide in 2021.^1^ Upon inhalation of aerosolized droplets containing M.tb, only a minority of people will progress to active tuberculosis (TB).^1^ The majority remain asymptomatic, having either cleared the infection or maintained a state of “latent” M.tb infection (LTBI). The clinical standard of care operationally defines LTBI as evidence of immune sensitization to M.tb, measured by positive tuberculin skin test (TST) or interferon gamma release assay (IGRA), without signs or symptoms of active TB. However, these tests do not distinguish the full spectrum of M.tb exposure and infection.^2^, ^3^, ^4^

Some individuals who are highly exposed to M.tb in community, household, and occupational settings maintain negative TST and IGRA results, which may reflect a state of enhanced immunologic protection from M.tb infection.^5^^,^^6^ We recently identified such “resisters” (RSTRs) among Ugandan household contacts with strong epidemiologic evidence of exposure to M.tb and showed that they harbored non-canonical T-cell and antibody responses to IGRA antigens ESAT-6 and CFP-10.^7^, ^8^, ^9^ However, most of the subjects in this cohort had only a single documented exposure occurring a median of 9 years prior to enrollment. A follow-up community-based study of highly exposed, HIV-infected, but TST- and IGRA-negative South Africans confirmed the presence of M.tb specific antibodies but did not evaluate T cell responses.^10^ Another study examined Chinese healthcare workers and identified protective antibody responses among both IGRA-positive and IGRA-negative study subjects.^11^ While these studies were conducted in areas with high TB endemicity, additional epidemiologic measurements of exposure were not determined. Thus, we sought to confirm and extend these results in a setting characterized by very intense infection pressure.

The prevalence of TB in South Africa is among the highest in the world, with an annual incidence of over 500 per 100,000 people.^1^ Men who work in gold mines represent one of the highest risk groups within South Africa, with an annual risk of M.tb infection as high as 20% per year, and case notification rates above 3000 per 100,000 miners, rivaling the rate of the pre-antibiotic era.^12^ HIV coinfection, exposure to silica dust, and close working and living conditions further predispose South African miners to active TB despite numerous public health interventions to mitigate the risk.^13^^,^^14^ Despite their long-term exposure in a high transmission setting, a cross-sectional study found that 13% of HIV-uninfected miners had zero millimeters of induration on TST,^15^ a definition thought to have 93% specificity for true clinical resistance to M.tb infection in this population.^16^

The recently reported Highly Exposed TB Uninfected (HETU) study sought to identify RSTRs, using a strict definition of negative IGRA result and TST induration of zero millimeters, among South African gold miners.^17^ No epidemiologic factors were identified that clearly distinguished RSTRs. However, miners who were of Black/African ethnicity, who were also more likely to work underground, were less likely to be RSTRs, indicating that epidemiology contributes to the risk of LTBI even within this highly exposed population. Nevertheless, 15% of all miners, including 11% of Black/African subjects, were identified as RSTRs in the HETU study.^17^

We used intracellular cytokine staining and systems serology to profile M.tb-antigen-specific T and B cell responses in matched RSTR and LTBI subjects identified through the HETU study. Among RSTRs, we detected both IFN-γ independent and, to a lesser extent, IFN-γ dependent T-cell responses. While RSTRs had detectable M.tb-specific IgG responses, these were at a lower level and more highly sialylated than those found in LTBI individuals. These data reveal adaptive immune signatures of M.tb exposure that are not detectable by routine clinical testing among a highly-exposed occupational cohort. Moreover, we found that T- and B-cell responses were associated via a TNF-mediated mechanism, pointing to the importance of this cytokine in mediating the immunologic response to M.tb in this highly and chronically exposed population.

## Methods

### Cohort and sample selection

The HETU study was conducted between July 2015 and December 2016 in the gold mines of Orkney in the North West province of South Africa.^17^ Enrolled individuals were 33–60 years old, had at least 15 years of employment in the mining industry, and were HIV-uninfected. All enrolees were male. QuantiFERON-TB Gold Plus assay (QFT-Plus) was performed at enrollment or within 90 days, and Mantoux tuberculin skin test (TST) was also performed within 7 days of QFT-Plus testing. Prior studies of this cohort defined RSTRs as individuals who had zero millimeters (mm) on initial TST and concordantly negative initial QFT-Plus test.^17^^,^^18^ For purposes of the current study, we were more stringent in our definition, requiring a second negative TST and QFT-Plus result at 12 months. LTBI individuals were defined as having positive QFT-Plus and TST tests at enrollment.

Blood was collected at enrollment, and peripheral blood mononuclear cells (PBMC) and serum were isolated and cryopreserved until use. For immunologic analysis, all RSTRs with available samples were included, and control LTBI samples were selected after matching for confounders, including age, body mass index (BMI), years worked in the mines, and ethnicity. A formal power calculation was not performed, but sample size was empirically informed by our prior study in Ugandan household contacts.^7^ Subjects analyzed in the current study were well matched except for an increased rate of Black/African ethnicity in LTBI individuals as noted in the overall cohort (Supplementary Table S1). In total, cryopreserved PBMCs and serum from RSTRs (n = 23 and 37, respectively) and LTBI subjects (n = 23 and 30, respectively) were analyzed. Correlations between T cells and antibody responses were determined using data overlapping between RSTRs (n = 21) and LTBIs (n = 9).

### Antigen selection

For T cell assays, overlapping peptide pools targeting ESAT-6 (BEI Resources Cat#NR-50711), CFP-10 (BEI Resources Cat#NR-50712), Ag85A (BEI Resources Cat#NR-34827), Ag85B (BEI Resources Cat#NR-34828), and TB10.4 (BEI Resources Cat#NR-34826) as well as H37Rv M.tb whole cell lysate (BEI Resources Cat#NR-14822) were used. Staphylococcal Enterotoxin Type B (SEB) (List Biological Laboratories Cat#122) was a positive assay control, and dimethyl sulfoxide (DMSO) (Sigma-Aldrich Cat#D8418) was a negative assay control.

For antibody assays, M.tb antigens tested were: purified protein derivative (PPD) (Statens Serum Institute), Ag85A and B in a 1:1 ratio (BEI Resources Cat#NR-49427 and #NR-53526), recombinant ESAT-6 (BEI Resources Cat#NR-49424) and CFP-10 (BEI Resources Cat#NR-49425) in a 1:1 ratio, HspX (BEI Resources Cat#NR-49428), 1-tuberculosyladenosine (1-TbAd) (provided by Dr. Branch Moody), and lipoarabinomannan (LAM) (BEI Resources Cat#NR-14848). An equal mixture of influenza antigens from HA1(B/Brisbane/60/2008) and HA1(H1N1) (A/New Caledonia/20/99) (Immune Technology Corp ITIT-003-001p and IT-003-B3p) was used as a positive assay control.

### Generation of tetramers

Lipid-loaded CD1 tetramers were generated as published previously.^19^ Briefly, glucose monomycolate (GMM) or synthetic diacylated sulfoglycolipid (Ac_2_SGL) was sonicated into pH 4.50 mM sodium citrate buffer, containing 0.25% 3-[(3-cholamidopropyl)dimethylammonio]-1-propanesulfonate (CHAPS). CD1b monomer was added to either a 20-fold (GMM) or 40-fold (Ac_2_SGL) molar excess of lipid. Monomer-lipid suspensions were incubated overnight at 37 °C and neutralized to pH 7.4 with 1 M Tris pH 9. Lipid-loaded monomers were tetramerized by adding 1.25 molar equivalents of fluorophore-conjugated streptavidin (Supplementary Table S2) at a 4:1 ratio of monomer to streptavidin at room temperature over 2 h then filtered through a SpinX column (Sigma-Aldrich Cat#CLS8162). Mock-loaded CD1b tetramers were generated by the same procedure without exogenous lipid. CD1d-PBS-57 (α-galactosylceramide or α-GalCer) PE and MR1-5-OP-RU BV421 were used as provided by the National Institutes of Health Tetramer Core Facility (Emory University, Atlanta, GA). Tetramers were validated by staining with a positive control T cell line.

### Flow cytometry

#### Intracellular cytokine staining (ICS)

PBMCs from RSTR (n = 23) and LTBI (n = 23) subjects were processed for intracellular cytokine staining as previously described.^7^ Briefly, samples were thawed and washed in RPMI 1640 with 10% fetal calf serum (FBS) and 2 μL/mL Benzonase (Millipore Cat#70746-3), then rested overnight in RPMI/10% FBS at 37 °C. The following day, PBMCs were stimulated for 6 h at 37 °C with peptide pools containing ESAT-6/CFP-10 or Ag85A/Ag85B/TB10.4, M.tb whole cell lysate, SEB, or 0.5% DMSO. In addition to antigen, each stimulation cocktail contained 1 μg/mL anti-CD28/49d (BD Biosciences Cat#347690), 10 μg/mL Brefeldin A (Sigma-Aldrich Cat#B7651), GolgiStop (BD Biosciences Cat#554724) prepared according to manufacturer's instructions, and anti-CD107a PE Cy7 (BD Biosciences Cat# 561348, RRID:AB_10644018). EDTA was added to a concentration of 2 mM to disaggregate cells. Then, samples were stored overnight at 4 °C before staining and acquisition by flow cytometry. We used a validated 11-color intracellular cytokine staining protocol (Supplementary Table S3). Cells were fixed in 1% paraformaldehyde in PBS and acquired on a BD LSRFortessa (BD Biosciences, San Jose, CA) equipped with a high-throughput sampler. Samples were processed in two batches in which the number of RSTRs and LTBI controls was matched, and the technician was not blinded to sample origin. No replicates for these samples were performed.

#### Combinatorial tetramer panel staining

To quantify the frequencies of donor-unrestricted T cell populations, we modified a previously validated assay designed to study *ex vivo* phenotypes of CD1b-restricted T cells.^19^ Briefly, cells were thawed and washed as above, then stained with Live/Dead Fixable Green Dead Cell Stain kit (Life Technologies Cat#L34970) at room temperature. Cells were blocked with human serum (Valley Biomedical Cat#HS1004CHI) diluted 1:1 with FACS buffer (0.2% bovine serum albumin in phosphate-buffered saline), before staining with CCR7 BV711 (BD Biosciences Cat# 566,602, RRID:AB_2739758) at 37 °C. To prevent internalization of the TCR upon tetramer binding, the FACS buffer was supplemented with the protein kinase inhibitor dasatinib (Cayman Chemicals Cat#11498) at 50 nM during the CCR7 and tetramer staining steps.^20^ Samples were washed and stained with one of two tetramer cocktails for 60 min at room temperature: one well was stained with a cocktail containing CD1d- α-GalCer PE tetramer, while the remaining wells were stained with a cocktail containing MR1-5-OP-RU BV421, CD1b-GMM APC, CD1b-GMM BV650, CD1b-Ac_2_SGL APC, CD1b-Ac_2_SGL ECD, and CD1b-Mock BV510 tetramers. After washing, samples were stained with an antibody cocktail (Supplementary Table S4) that was supplemented with 1 mM l-ascorbic acid and 0.05% sodium azide to reduce cell-mediated fluorophore degradation. Samples were washed twice with PBS, and fixation and acquisition were performed as described above. A positive control was generated by mixing PBMC from an irrelevant donor with polyclonal T cell lines specific for CD1b-GMM CD1b-Ac_2_SGL.^19^ Researchers were not blinded to sample origin.

#### Data analysis

Raw flow cytometry data was compensated and manually gated using FlowJo v9.9 (TreeStar Inc, Ashland, OR), and data was processed using the OpenCyto framework in the R programming environment.^21^ Samples with poor viability, defined by low CD3 counts (<10,000 cells) or low CD4 counts (<3000 cells), were excluded from further analysis. Final data for ESAT-6/CFP-10 and Ag85A/Ag85B/TB10.4 included 22 RSTRs and 19 LTBI controls; for M.tb lysate, 21 RSTRs and 20 LTBI; for SEB, 22 RSTRs and 20 LTBI controls. To assist with setting gates for the tetramer panel, fluorescence minus one (FMO) controls were run for each tetramer alongside the fully stained control. Boolean gating was used to identify events that were specific for each tetramer. The final analysis includes all 23 RSTR and 23 LTBI control samples.

To analyze which T-cell subsets were activated by antigen stimulations, we used COMbinatorial Polyfunctionality Analysis of Antigen-Specific T cell Subsets (COMPASS),^22^ which uses a Bayesian computational framework to identify T-cell subsets for which there is a high probability of antigen-specific response. COMPASS compares the proportion of events in the antigen-stimulated sample to the proportion of gated events in the control sample. For data presented here, COMPASS was applied to each of the five stimulations of CD4^+^ T cells. Each one of the analyses was unbiased and considered all of the 128 possible cytokine functions (defined as Boolean combination). For a given subject, COMPASS was also used to compute a functionality score (FS) that summarizes the entire functional profile into a single continuous variable that can be used for standard statistical modeling (e.g., regression).

### Customized multiplex luminex assay

A Luminex assay was used to quantify the relative levels of antigen-specific antibody isotypes and subclasses, their ability to bind Fc receptors, and their Fc glycosylation patterns. Luminex Magplex carboxylated microspheres (Luminex Corporation) were coupled to proteins via covalent N-hydroxysuccinimide (NHS)–ester linkages by 1-ethyl-3-(3-dimethylaminopropyl)carbodiimide hydro-chloride (EDC) and sulfo-NHS per manufacturer recommendations. LAM and 1-TbAd were modified by 4-(4,6-dimethoxy [1,3,5]triazin-2-yl)-4-methyl-morpholinium (DMTMM) prior to conjugation.

Assays were optimized over a dilution curve to ensure selection of a dilution within the linear range of the assays. A 1:200 dilution was selected to maximize the dynamic range across control samples and to capture the area under the curve for the full range of dilutions tested. Diluted serum samples were incubated with pooled microspheres for 16 h at room temperature then washed three times with 0.1% bovine serum albumin (BSA)/0.05% Tween in PBS. Secondary incubations were performed for 2 h at room temperature (Supplementary Table S5). Then, samples were washed three times prior to acquisition. For each assay, median fluorescence intensity (MFI) for each bead region was measured using an iQue Plus Screener (Intellicyt). Lectin binding measurements were normalized to total antigen-specific IgG. All samples were assayed in duplicate, and values were averaged. Experimenters were blinded to sample identification.

### Statistics

All univariate comparisons of RSTR and LTBI groups for T-cell and antibody data were performed with Mann–Whitney U test. Corrections for multiple hypothesis testing was performed using the Benjamini-Hochberg method.

### Multivariate modeling of T- and B-cell responses

Multivariate signatures of RSTR and LTBI status were developed for overlapping individuals in the combined COMPASS and antibody datasets as described above.^23^ Since IFN-γ release defines RSTR and LTBI phenotypes, ESAT-6/CFP-10 specific IFN-γ containing T-cell subsets were removed from the dataset. MFI values were log_10_ transformed and all data were then Z-scored. Least absolute shrinkage and selection operator (LASSO)^24^ regression was used to select the minimal antibody and T-cell features that captured the variation between the groups using R package “glmnet” with default parameters, as we have done previously.^7^ To estimate the minimal correlates that best explained group differences without overfitting, a five-fold nested validation framework was designed. In each repetition, the dataset was divided into groups of randomly assorted individuals, where 80% of the dataset was used for building the model and the remaining holdout set was used to test the model prediction. The goodness-of-fit of the model was measured by classification accuracy between RSTRs and LTBI. At each LASSO run, lambda parameter was optimized using *cv.glmnet* function. Ultimately, this approach resulted in the generation of a model with the minimal set of features that generated the best classification prediction in a cross-validation test. Partial least squares Discriminant Analysis (PLS-DA) was then used to visualize antibody profiles using these minimal LASSO-selected features in multivariate space, using package “ropls.”^25^

Correlation networks were created using co-correlates of all LASSO-selected features. Correlations were performed using Spearman method followed by Holm correction, and all correlates with Benjamini-Hochberg corrected p value of <0.05 were included. The co-correlate network was generated using R package “network.”^26^ All multivariate analyses were performed in R version 1.16.0.^27^

### Ethics

The HETU study (protocol AUR2-7-160) was approved by the ethics committee of the University of Witwatersrand (reference number 150217), as well as the London School of Hygiene and Tropical Medicine, the University of Washington, and Massachusetts General Hospital. All participants gave written informed consent, or if illiterate, gave verbal consent with an impartial witness.

### Role of the funding source

No study sponsor was involved in decisions regarding the collection, analysis, interpretation of data, the writing of the manuscript, or the decision to submit for publication.

## Results

### Highly exposed TB uninfected (HETU) South African gold miners

We aimed to study immune profiles in matched RSTR and LTBI subjects identified through the HETU study, a longitudinal cohort of South African gold miners.^17^ Briefly, individuals who had worked for at least 15 years in the mining industry were designated as LTBI on the basis of a positive IGRA and at least five millimeters (mm) of induration on TST at enrollment. RSTRs were defined by having both a negative IGRA and zero mm of induration on TST at enrollment. To enrich for a stable clinical phenotype, we further refined the definition of RSTR to exclude those who had either a positive QFT-Plus test or TST induration >5 mm at the 12-month visit. A subset of RSTR (n = 23) and LTBI (n = 23) individuals were selected for M.tb-specific T cell profiling on the basis of matching for age, body mass index, and years worked in the mines, and a second subset of 37 RSTR and 30 LTBI individuals were selected for serum M.tb-specific antibody profiling (Supplementary Table S1). The overlapping 21 RSTR and 9 LTBI individuals were available for analysis of both B and T cell responses. Black/African ethnicity, which was associated with RSTR status in the larger HETU study, was also significantly associated with RSTR status in the subset of individuals included here (Supplementary Table S1).

### RSTRs demonstrate IFN-γ independent T cell responses targeting ESAT-6 and CFP-10

Peripheral blood mononuclear cells (PBMCs) were stimulated with overlapping peptide pools targeting ESAT-6/CFP-10 and assessed for seven T cell functions using a previously validated intracellular cytokine staining (ICS) assay, capturing distinct T cell functional subsets producing interleukin (IL)-2, IL-4, IL-17a, IFN-γ, tumor necrosis factor (TNF) and CD107a, and expressing CD40L/CD154 (Supplementary Fig. S1a).^28^^,^^29^ COMbinatorial Polyfunctionality Analysis of Antigen-Specific T cell Subsets (COMPASS) was used to ensure the detection of even rare cytokine-producing T cell populations.^22^

**Fig. 1:**
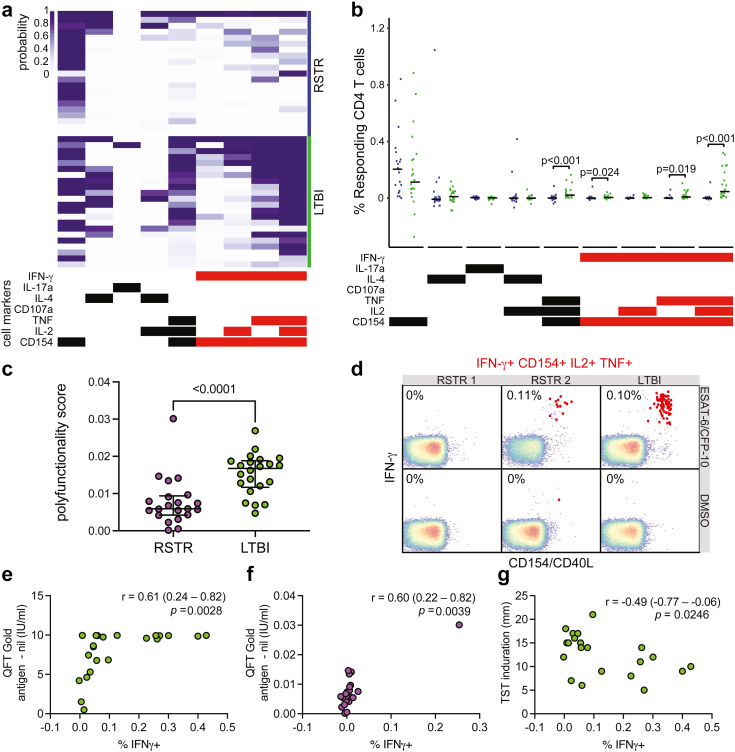
**RSTRs demonstrate IFN-γ independent T cell responses targeting ESAT-6 and CFP-10.** (a) ICS data was generated from PBMCs from RSTR (n = 23) and LTBI (n = 23) subjects from the HETU study in response to stimulation with ESAT-6/CFP-10 peptide pool and analyzed by COMPASS. In the heatmap, rows represent study subjects, and columns represent CD4 T cell functional subsets. The depth of shading reflects the probability of detecting a response above background. CD4 subsets are indicated by bars at the bottom, with IFN-γ containing subsets shown in red. (b) Background corrected magnitudes (peptide pool minus DMSO) for each of the subsets identified by COMPASS are shown for RSTR (purple) and LTBI (green) individuals. Groups were compared by Mann–Whitney U test, and nominal p values < 0.05 are labeled. (c) Subject-specific COMPASS results were summarized for RSTR and LTBI individuals using the polyfunctionality score, which weights T cell subsets that include more than one function. Medians and interquartile ranges are depicted. The statistical significance was calculated using the Mann–Whitney U test, and the two-tailed p value is indicated. (d) Representative flow cytometry plots from two RSTR and one LTBI control subject are shown examining an IFN-γ-containing T cell subset in response to stimulation with DMSO or ESAT-6/CFP-10. Frequencies of T cells simultaneously producing IFN-γ, CD154, TNF, and IL-2 are shown and indicated as red dots in the typical two-dimensional layout. (e) Percentage of IFN-γ+ T cells for each subject was directly plotted against quantitative QFT result at baseline for LTBI subjects. (f) Percentage of IFN-γ+ T cells for each subject was directly plotted against quantitative QFT result at baseline for RSTRs with Spearman correlation coefficients (r) and p values indicated. (g) Percentage of IFN-γ+ T-cells was plotted against TST diameter at baseline for LTBI subjects. Spearman correlation coefficient and p value is indicated.

As expected, LTBI subjects demonstrated both IFN-γ dependent and IFN-γ independent T cell responses to ESAT-6 and CFP-10 (Fig. 1a). RSTRs displayed a similar magnitude of IFN-γ independent T cell responses to that seen in LTBI subjects, including expression of CD154 (Fig. 1b). Surprisingly, some RSTRs showed evidence of IFN-γ positive immunity as well, though these responses were of lower magnitude than those of LTBI subjects (Fig. 1a and b). Overall, polyfunctionality scores (PFS) were also almost three-fold lower among RSTRs than LTBIs, largely due to the lack of IFN-γ dependent immunity (Fig. 1c). However, the range of responses among RSTRs varied, with at least one participant displaying responses that were indistinguishable from LTBIs and another showing no detectable response at all (Fig. 1d).

The presence of IFN-γ positive responses among RSTRs was inconsistent with their two negative IGRA results over 12 months. We ruled out several technical explanations for this result, including cross-contamination of stimuli or cells, sample mislabeling, and errors in gating. To further explore a biological basis for these results, we next examined the correlation between the clinical assessment of IFN-γ production by IGRA and the total frequency of IFN-γ containing subsets in the COMPASS dataset. Indeed, we observed a modest correlation (r = 0.61 (95% confidence interval 0.24–0.82; p = 0.0028 [Spearman correlation]) among LTBI subjects (Fig. 1e). The RSTRs all had QFT results below the positivity threshold of 0.35 IU/mL established by the manufacturer,^30^ but the distribution of IFN-γ frequencies determined by flow cytometry in RSTRs nevertheless overlapped markedly with those seen in LTBI. A correlation between RSTRs’ QFT results and IFN-γ was detectable (r = 0.60 (95% confidence interval 0.22–0.82), p = 0.0039 [Spearman correlation]) (Fig. 1f), driven by one individual with the highest probability of IFN-γ by COMPASS who also had the highest result on QFT testing. A weak negative correlation was instead observed between TST induration and IFN-γ frequency among LTBI subjects (r = −0.49 (95% confidence interval −0.77 to −0.06, p = 0.0246 [Spearman correlation]) (Fig. 1g), perhaps due to TST responses to antigens other than ESAT-6/CFP-10. Thus, South African RSTRs displayed a spectrum of T cell responses to ESAT-6 and CFP-10, ranging from no detectable cytokine producing cells to IFN-γ independent and even some low level IFN-γ positive T-cell responses.

### RSTRs demonstrate reduced functional breath to cross-reactive mycobacterial antigens

**Fig. 2:**
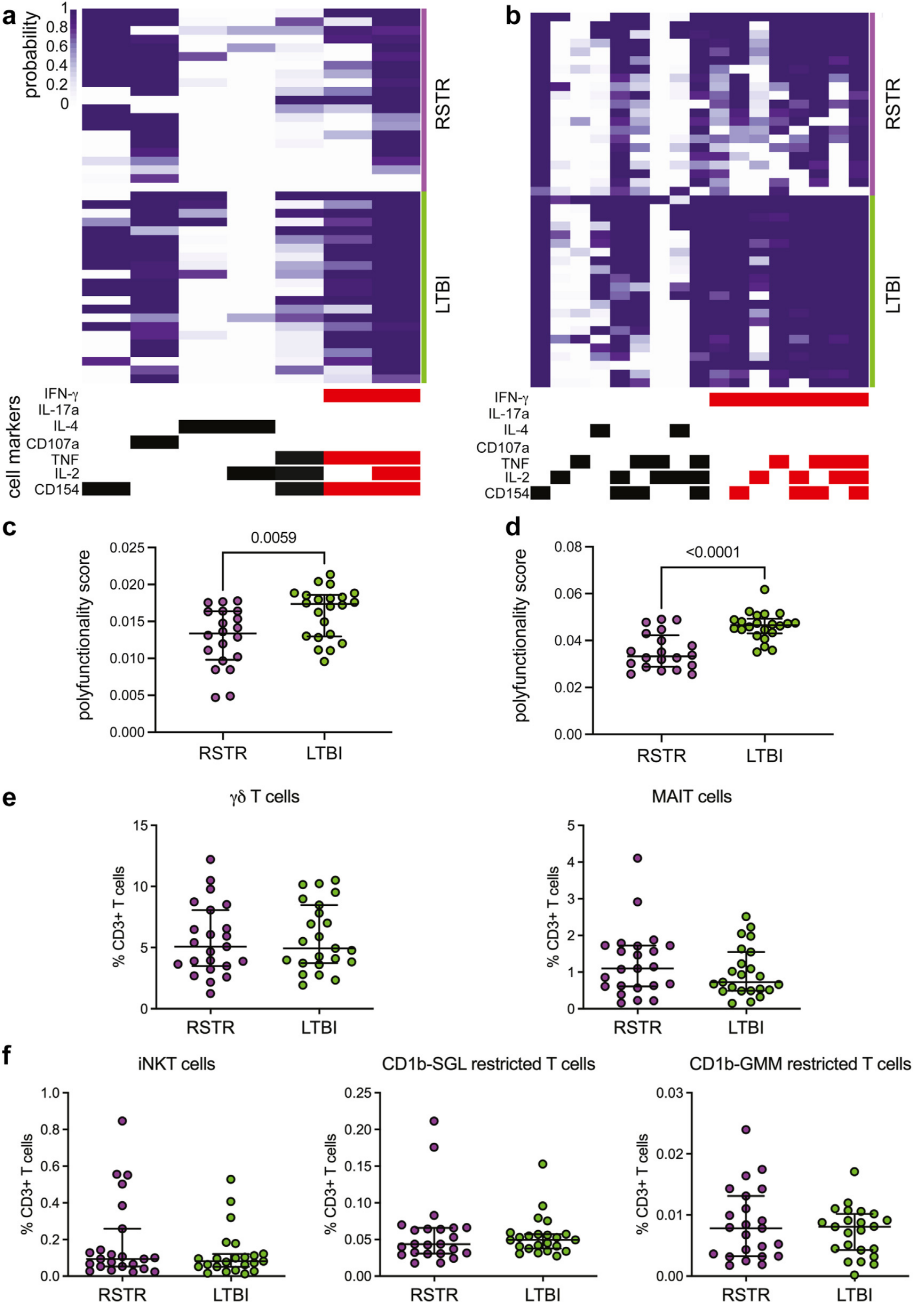
**RSTRs demonstrate reduced functional breath to cross-reactive mycobacterial antigens.** COMPASS was used to analyze ICS data from RSTRs (n = 23) and LTBI (n = 23) subjects in response to stimulation with (a) Ag85A, Ag85B, and TB10.4 peptide pool or (b) M.tb lysate. In the heatmap, rows represent study subjects, and columns represent CD4 T cell functional subsets. The depth of shading reflects the probability of detecting a response above background. CD4 subsets are indicated by bars at the bottom, with IFN-γ containing subsets shown in red. COMPASS results for stimulation with (c) Ag85A, Ag85B, and TB10.4 peptide pool or (d) M.tb lysate were summarized for RSTR and LTBI individuals using the polyfunctionality score. Medians and interquartile ranges are depicted. The statistical significance was calculated using the Mann–Whitney U test, and the two-tailed p value is indicated. Combinatorial tetramer staining and multi-parameter flow cytometry was used to quantify frequencies of donor-unrestricted T cells. (e) Frequencies of γδ T cells or mucosal-associated invariant T (MAIT) cells as a proportion of live CD3+ T-cells are displayed as boxplots with the median and interquartile range indicated. Statistical testing was performed using the Mann–Whitney U test. (f) Frequencies of invariant NKT (iNKT), as well as CD1b-sulfoglycolipid (CD1b-SGL) and CD1b-glucose monomycolate (CD1b-GMM) restricted T cells as a proportion of live CD3+ T-cells are displayed as boxplots with the median and interquartile range indicated. Groups were compared using the Mann–Whitney U test.

We next examined T cell responses to cross-reactive mycobacterial antigens, antigen 85 (Ag85) A and B, TB10.4, and M.tb lysate (Fig. 2a and b). COMPASS revealed IFN-γ positive T-cell responses to these stimulations in both RSTR and LTBI individuals. However, the overall PFS remained higher in LTBIs compared to RSTRs, largely as a result of IFN-γ positive responses (Fig. 2c and d). We also explored whether donor-unrestricted T cells, including T cells targeting mycobacterial lipid antigens glucose monomycolate (GMM) and diacylated sulfoglycolipid (Ac_2_SGL), were associated with “resistance” to M.tb infection. We used combinatorial tetramer staining and multi-parameter flow cytometry to quantify the frequencies of donor-unrestricted T-cells in RSTRs (n = 23) and LTBIs (n = 23) (Supplementary Fig. S1b).^19^ We observed no difference in circulating frequencies of γδ T cells, iNKT cells, MAIT cells, GMM-specific, or Ac_2_SGL-specific T cells between RSTRs and LTBI controls (Fig. 2e and f). Together, these data show that South African miner RSTRs and LTBI both exhibit IFN-γ-dependent and independent responses to cross-reactive mycobacterial antigens.

### M.tb-specific antibody profiles distinguish RSTRs from LTBI subjects

We next used systems serology to determine whether distinct M.tb-specific antibody profiles could be found in RSTRs compared with LTBI individuals.^31^, ^32^, ^33^ Serum was analyzed from RSTR (n = 37) and LTBI (n = 30) subjects (Supplementary Table S1). We used a customized multiplex Luminex assay to characterize serum antibody responses to a panel of M.tb antigens including PPD, ESAT-6/CFP-10, Ag85, HspX, and 1-TbAd. For each individual's peripheral antigen-specific response, we measured levels of total IgG and IgG subclasses IgG1-IgG4, IgA1 and IgA2, and IgM, binding of Fcγ receptors 2A, 2B, 3A, and 3B, IgG Fc sialylation, and IgG Fc galactosylation (Supplementary Fig. S2).

**Fig. 3:**
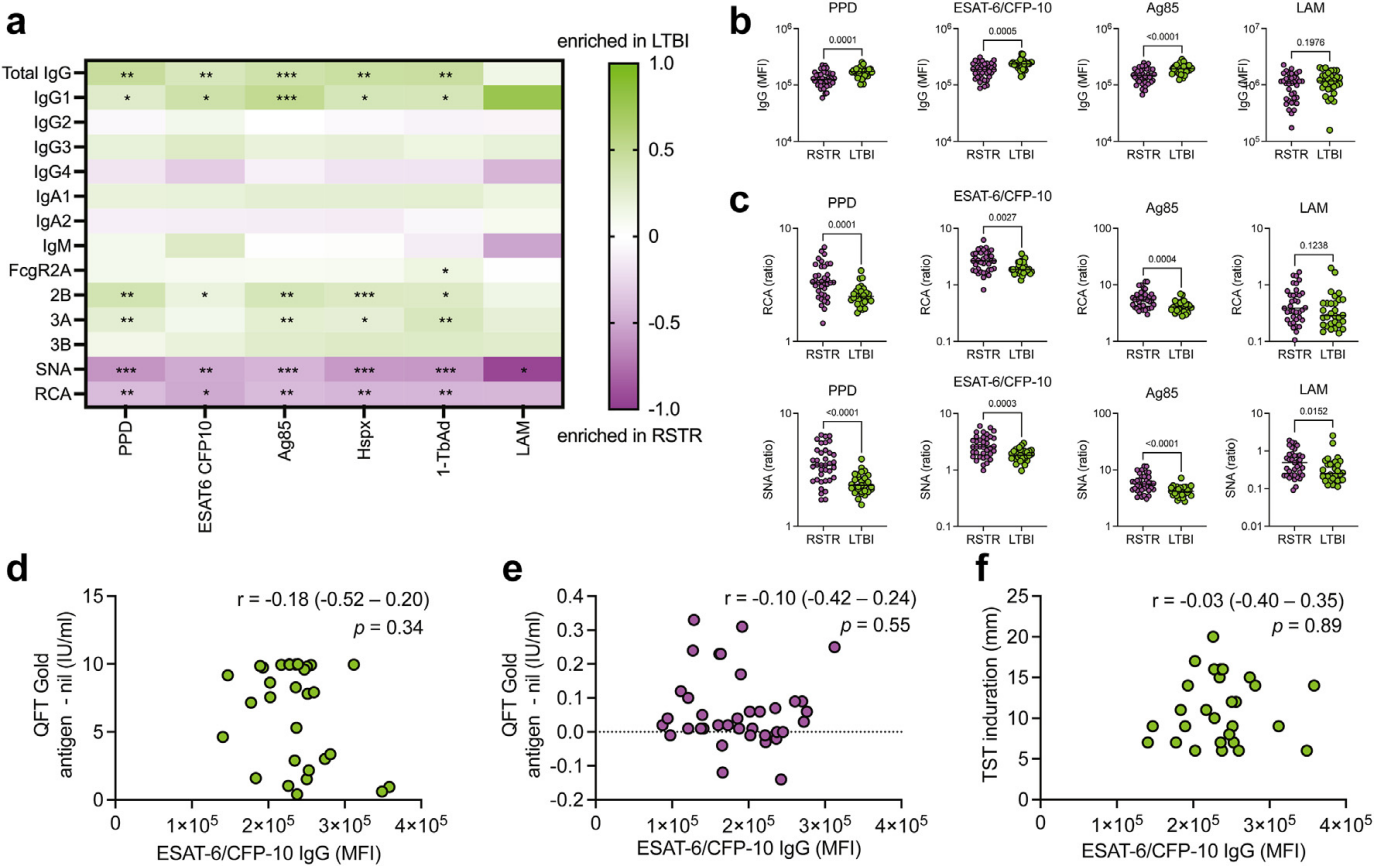
**M.tb-specific antibody profiles distinguish RSTR from LTBI individuals.** (a) A customized, multiplex Luminex assay was used to compare the isotype, subclass, FcγR binding, and Fc glycosylation of antibodies targeting a panel of M.tb antibodies in RSTRs (n = 37) and LTBI (n = 30) subjects. For each assay, the heatmap color indicates log_2_ [(median value in LTBI)/(median value in RSTRs)]. Groups were compared using Mann–Whitney U test, and p values are shown for those that remain significant after Benjamini-Hochberg test. ∗p < 0.05, ∗∗p < 0.01, ∗∗∗p < 0.001. (b) Subject-specific univariate comparisons are shown for IgG level. p values assessed by Mann–Whitney U test are shown. Dotted line indicates median value. (c) Subject specific univariate comparisons are shown for Fc galactosylation (measured by RCA binding) and Fc sialylation (measured by SNA binding) of antibodies targeting PPD, ESAT-6/CFP-10, Ag85, and LAM. Dotted line indicates median value. (d) ESAT-6/CFP-10 IgG levels were plotted against quantitative QFT result at baseline for LTBI subjects. (e) ESAT-6/CFP-10 IgG levels were plotted against quantitative QFT result at baseline for RSTRs. (f) ESAT-6/CFP-10 IgG levels were plotted against TST induration at baseline for LTBI subjects. For (d)–(f), Spearman correlation coefficient, r, and p value are shown on each plot.

Both RSTR and LTBI subjects had detectable M.tb-specific antibody responses with largely overlapping distributions, but LTBI individuals had modestly higher levels of IgG and IgG1 specific to almost all tested M.tb antigens (PPD, ESAT-6/CFP-10, Ag85, HspX, and 1-TbAd). LAM-specific IgG and IgG1 also trended higher in LTBI subjects (Fig. 3a, with selected univariate plots shown in Fig. 3b). Additionally, LTBIs had increased FcγR binding across almost all tested antigens, particularly of FcγR2B and FcγR3A (Fig. 3a). Conversely, RSTRs had higher IgG Fc sialylation and galactosylation across all tested M.tb antigens, with only LAM-specific Fc galactosylation not achieving statistical significance (Fig. 3a, with selected univariate plots shown in Fig. 3c).

Finally, we asked whether ESAT-6/CFP-10 antibody responses correlated with the IFN-γ responses detected by IGRA. ESAT-6/CFP-10-specific IgG responses in LTBI and RSTRs had high variance and exhibited overlapping distributions but did not correlate with IFN-γ secretion measured by IGRA (Fig. 3d and e) or with TST induration (Fig. 3f). Taken together, these results reveal unique M.tb-specific antibody features that are preferentially associated with RSTR or LTBI status and highlight Fc glycosylation as a potential correlate of “resistance” to M.tb.

### Combinations of T cell and antibody features accurately classify RSTRs and LTBI subjects

**Fig. 4:**
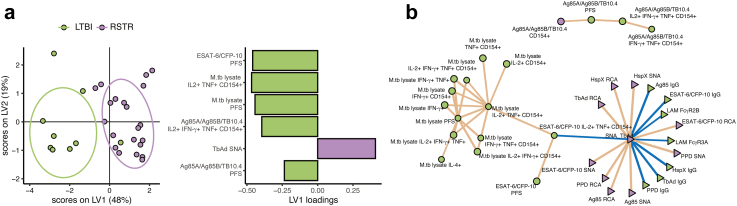
**RSTRs and LTBI subjects have distinct M.tb-specific adaptive immune profiles.** (a) LASSO was applied to the full COMPASS and Luminex dataset for overlapping individuals to select a minimal set of features that best captured the variation between RSTR (n = 21) and LTBI (n = 9) subjects. Partial least squares discriminant analysis (PLS-DA) was then used to visualize the individuals in multivariate space captured by LASSO-selected parameters. Loadings plot indicates the relative contributions of each of the seven LASSO-selected features to the latent variable 1 (LV1, x-axis). (b) Correlation networks were developed between LASSO-selected features and all remaining features. All correlations were plotted with p value of <0.05 after Benjamini-Hochberg correction. T-cell measurements are shown with circles, and antibody measurements with triangles. Color of each feature represents relative enrichment in RSTRs (purple) versus LTBI (green). Positive correlations are shown with brown lines and negative correlations with blue lines.

We asked whether the combination of all measured antigen-specific T cell and B cell responses could define distinct immune profiles for RSTR and LTBI individuals. We used the least absolute shrinkage and selection operator (LASSO)^24^ to select the minimal set of features that best captured the variance separating the two groups and partial least squares discriminant analysis (PLS-DA) to create a multivariate map of the individuals along the LASSO-selected variables (Fig. 4a). Because ESAT-6/CFP-10 specific IFN-γ production defined RSTR and LTBI status, all IFN-γ dependent responses to ESAT-6/CFP-10 were excluded from the model. Using only six features, the model was able to separate RSTRs from LTBI subjects. Of these six features, five were T-cell based, including PFS against all three stimulations, IL-2+ IFN-γ+ TNF + CD154+ subsets stimulated by Ag85A/Ag85B/TB10.4, and IL-2+ TNF + CD154+ subsets stimulated by M.tb lysate. A single antibody feature was also selected: 1-TbAd-specific Fc sialylation. All features were enriched in LTBI over RSTR individuals except for 1-TbAd-specific Fc sialylation, which was enriched in RSTRs.

Since LASSO selects a minimal set of features that best differentiate groups, the algorithm excludes variables that correlate closely with those that are selected. To more fully understand the patterns of immune responses that differentiated LTBI and RSTR individuals, we therefore examined a network of measured features that correlated with those selected by LASSO (Fig. 4b). Within the T cell data, we observed correlation networks that largely clustered on the basis of stimulation condition, with prominent presence of subsets containing CD154 and TNF as key T-cell features differentiating LTBI from resisters. On the other hand, correlations within the antibody data were clustered on the basis of isotype, rather than antigen, with a large network including IgG, Fc sialylation, and Fc galactosylation against multiple antigens. Thus, unlike T-cell responses, antibody responses were coordinated across M.tb antigens. Further, increased IgG, decreased Fc sialylation and galactosylation, and increased FcR binding were the most important antibody features that differentiated RSTRs from LTBI subjects. Taken together, these data reveal that patterns of correlated M.tb-specific T cell and antibody responses are able to fully differentiate RSTR and LTBI subjects.

### Antigen-specific TNF- producing T cells correlate with circulating IgG levels

**Fig. 5:**
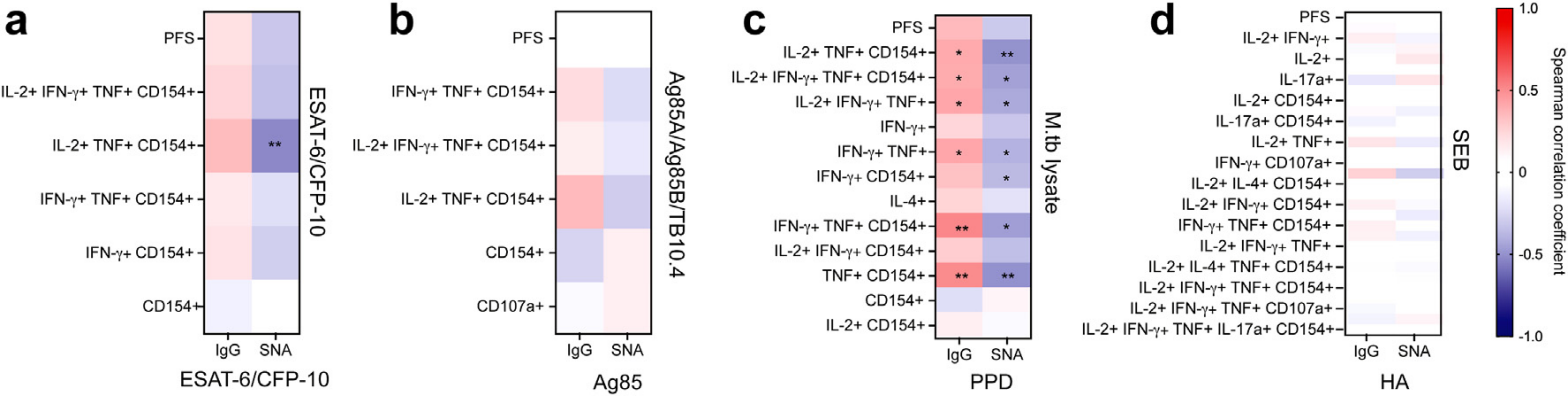
**Antigen-specific TNF-producing T cell subsets correlate with circulating IgG levels.** (a) Spearman correlations were assessed between ESAT-6/CFP-10 specific COMPASS subsets and PFS and ESAT-6/CFP-10-specific IgG level and sialylation, for RSTR (n = 21) and LTBI (n = 9) subjects overlapping between COMPASS and Luminex datasets. Color of heatmap indicates degree of correlation coefficient from −1 to 1. ∗p < 0.05, ∗∗p < 0.01. (b) correlations were measured between Ag85A/Ag85B/TB10.4 -specific COMPASS subsets and Ag85-specific IgG level and sialylation. (c) Correlations were assessed between M.tb lysate specific COMPASS subsets and PPD-specific IgG level and Fc sialylation. (d) Correlations were assessed between Staphylococcal enterotoxin B-specific COMPASS subsets and influenza hemagglutinin-specific IgG level and Fc sialylation.

Having identified the presence of both T and B cell responses targeting M.tb in RSTRs, we next asked whether antigen-specific T- and B-cell responses were correlated with each other among subjects who had been analyzed in both datasets. We first investigated whether the frequency of any ESAT-6/CFP-10-specific T cell subsets correlated with the ESAT-6/CFP-10-specific IgG levels and degree of Fc sialylation (measured by SNA), by calculating Spearman correlation coefficients for each comparison (Fig. 5a). Nearly all correlations were not significant, with only a single negative correlation, between SNA and IL-2+TNF+CD154+ T cells, achieving statistical significance (r = −0.53, p = 0.003 [Spearman correlation]). Ag85A/Ag85B/TB10.4-specific T cell subsets and Ag85 complex-specific antibody responses were weakly correlated (Fig. 5b). Finally, to investigate antigens not specifically included in either pool, we compared M.tb lysate-stimulated T cell subsets to PPD-specific antibody IgG and Fc sialylation (Fig. 5c). All TNF- containing subsets demonstrated modest but statistically significant positive correlations with levels of PPD-specific IgG and negative correlations with SNA binding to PPD-specific IgG. In contrast, T cell subsets resulting from stimulation of the positive control staphylococcal enterotoxin type B (SEB) did not correlate with the positive antibody control nor IgG to influenza hemagglutinin (HA), indicating the observed correlations were in fact M.tb-specific (Fig. 5d). These data indicate a potential role for TNF in coordinating responses between M.tb specific T- and B-cells.

## Discussion

In recent years, several reports have identified a phenotype of “resistance” to M.tb infection in a variety of geographic settings and implicated a wide range of innate and adaptive immune mechanisms.^8^^,^^11^^,^^34^, ^35^, ^36^, ^37^, ^38^, ^39^, ^40^ Our prior work focused on Ugandan household contacts with a distant history of intense exposure to M.tb who remained persistently IGRA and TST negative while continuing to live in an endemic area. Among those RSTRs, we demonstrated M.tb-specific IFN-γ-independent T-cell and antibody responses consistent with their epidemiology.^7^ Here, we extended these findings in a cohort of South African gold miners characterized by chronic, high-intensity exposure to M.tb. Again, we identified within RSTRs the presence of IFN-γ independent T cell and antibody responses to M.tb-specific antigens ESAT-6 and CFP-10. However, we also discovered distinct antibody profiles among RSTRs, including markedly increased IgG Fc sialylation. Finally, we discovered a consistent relationship between M.tb-lysate specific T cell subsets producing TNF and PPD-specific antibody responses, suggesting that TNF may mediate interactions between M.tb-specific T cells and B cells. In summary, our data validate and extend our original observation to an occupational cohort characterized by intense and long-term infection pressure and reveal potential mechanisms underlying “resistance” to M.tb infection in humans.

Population-based studies, as well as studies of genetically diverse mouse models, suggest the presence of an immunologic spectrum of risk, in which “resistance” to M.tb infection can be overcome with sufficient exposure.^41^, ^42^, ^43^ Consistent with this hypothesis, COMPASS analysis revealed the presence of IFN-γ positive T cell responses to ESAT-6 and CFP-10 in a minority of RSTRs. Notably, these responses were generally of low magnitude and near the threshold for detection, illustrating the sensitivity of the COMPASS algorithm.^22^ These results contrast with our prior study of Ugandan household contacts, where we did not detect IFN-γ positive responses among RSTRs. This difference potentially reflects differences in burden of M.tb exposure between the two populations, suggesting enrichment for true clinical resistance in South Africa that may be represented by the few RSTRs who had almost no detectable ESAT-6/CFP-10-specific T cell responses (Fig. 1a). However, there are alternative explanations for these seemingly conflicting results. Ugandan RSTRs were defined stringently by longitudinal follow up over a median of 9.5 years and concordantly negative results on seven TST and three IGRA tests.^8^^,^^9^ South African RSTRs were only tested at enrollment and at 12 months, so may have included individuals with missed or delayed IGRA conversion and/or reversion.^17^^,^^44^ Additionally, immune responses of the Ugandan individuals were measured several years after their single documented exposure.^7^^,^^8^ In contrast, South African miners had ongoing M.tb exposure at sample collection that may be reflected in their immune signatures.^45^

In both Uganda and South Africa, we observed that multiple M.tb-specific T-cell responses, including polyfunctionality scores to all tested stimulations, were greater among LTBI controls compared to RSTRs (Fig. 1, Fig. 2). Conversely, while M.tb antibody responses were similar among Ugandan RSTRs and LTBIs, in South Africa we identified differences in RSTRs' antibody profiles, including decreased IgG responses and increased Fc sialylation and galactosylation (Fig. 3a–c). Terminally sialylated glycans are essential for anti-inflammatory effects of antibodies,^46^ suggesting that RSTRs may harbor an anti-inflammatory humoral phenotype. These differences from our Uganda study again support the critical impact of epidemiology on individuals’ M.tb-specific immune responses.^17^ The clear evidence of M.tb immune sensitization found in RSTRs in both cohorts, however, emphasizes that antibody responses and IFN-γ independent T cell responses may identify M.tb exposure that is not reflected by traditional diagnostic testing. An examination of M.tb specific adaptive immune responses in low risk, IGRA-negative individuals may help to elucidate more specific immune markers of M.tb exposure.

The results reported here validate our original observation that IFN-γ independent T cell responses to ESAT-6 and CFP-10 are characterized by robust expression of CD154/CD40L, which we proposed may mediate the induction of humoral immunity given its role in generating high-affinity, class-switched antibody responses.^7^^,^^47^ We extended our original results by performing a correlation analysis across all T cell and antibody features and discovering that M.tb lysate-specific TNF + T-cell subsets were positively correlated with PPD-specific IgG, and negatively correlated with Fc sialylation. TNF and the TNF receptor superfamily play critical roles in B cell maturation, proliferation, and function.^48^, ^49^, ^50^, ^51^ Notably, we recently reported transcriptomic evidence of increased TNF signaling in M.tb-infected monocytes from South African RSTRs compared to LTBI subjects,^18^ although we did not observe increased TNF secretion by RSTR T-cells in the current study (Fig. 1, Fig. 2). Collectively, these data identify TNF as a soluble mediator of a possible second cognate interaction between M.tb-specific T cells and B cells, resulting in increased production of M.tb-specific IgG.

Major strengths of this study are the unique occupational cohort with a high burden of TB exposure and stringent definition of RSTRs, which have afforded us an unprecedented opportunity to explore the T- and B-cell adaptive immune responses that underlie M.tb “resistance” in this context. Nevertheless, an important limitation is the possible contribution of unmeasured epidemiologic confounders to the differential immune signatures observed. In the HETU study, LTBI status was associated with Black/African ethnicity,^17^ which was also associated with underground work and likely unmeasured social and economic factors that contribute to higher rates of M.tb infection. However, this makes the immunologic evidence of M.tb exposure found here in the relatively lower risk RSTRs all the more striking. A second important caveat is that South African miners have an increased rate of infection with *M. kansasii*,^14^ one of the few non-tuberculous mycobacterial species that also produce the antigens ESAT-6 and CFP-10.^52^ The estimated rate of *M. kansasii* infection among miners, however, is so much lower than that of M.tb^12^ that we believe the observed adaptive responses largely reflect exposure to M.tb itself.

The identification of highly exposed individuals who show no clinical evidence of M.tb infection represent a valuable opportunity to uncover protective immune correlates from M.tb infection. Our results, obtained in the context of a highly and chronically exposed cohort of South African miners, support and extend a growing field of data that RSTRs harbor coordinated, M.tb-specific adaptive immune responses that are typically missed by standard clinical assays. Probing the immunologic mechanisms that define protective immunity in such populations may be the key to developing novel treatments to curb the TB epidemic.

## Contributors

LRLD, LL, CS, GA, and SMF conceived the study. LRLD, CS, and GA wrote the manuscript with contributions from all authors. EDL performed or supervised all flow cytometry experiments and collected the data. Flow cytometry data were accessed and verified, analyzed, and interpreted by MTS, EDL, and CS. LRLD, SF, and JSL performed all antibody experiments and collected the data. Antibody data were accessed and verified, analyzed, and interpreted by LRLD and GA. DC performed multivariate LASSO PLS-DA analysis and correlation network analysis. GJC, TRH, RSW, ADG, and KF were responsible for the conceptualization and methodology of the HETU Study, and GJC and RSW facilitated access to biologic samples. CMS, WHB, and TRH provided funding and oversight for the work. All authors read and approved the final manuscript.

## Data sharing statement

All raw antibody and COMPASS data generated in this study, as well as metadata for all subjects, are included in a supplementary data file.

## Declaration of interests

Galit Alter became an employee of Moderna Therapeutics after completion of the work described here and holds equity in Leyden Labs and Systems Seromyx.
