# Supplementary material for: IFN-γ independent markers of Mycobacterium tuberculosis exposure among male South African gold miners
Source: eBioMedicine. 2023 Jun 26;93:104678. doi: 10.1016/j.ebiom.2023.104678 (PMC10320233; doi:10.1016/j.ebiom.2023.104678)
Supplement: Supplementary Fig. S1 [file mmc1.pdf]

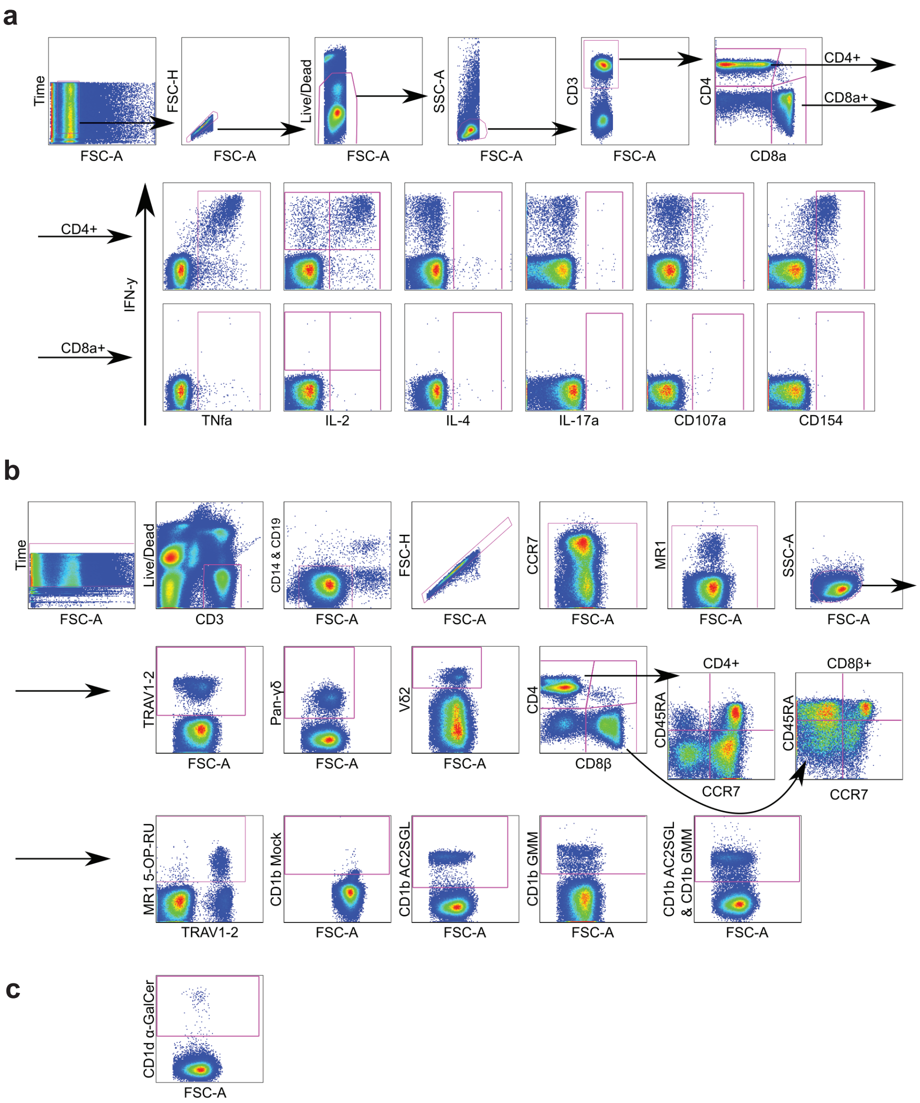

Supplementary Figure 1. Gating strategy for T cell flow cytometry. (A) Data obtained using an 11-color multiparameter intracellular cytokine staining (ICS) panel were gated as shown in a representative patient sample stimulated with M.tb lysate. Events acquired during steady flow were isolated using a time gate, followed by a singlet gate. Viable cells were identified, and the lymphocytes were selected based upon their relative size and complexity. T cells were identified by staining with CD3, and the T cells were subdivided based upon CD4 and CD8a co-receptor expression. Each population was characterized for expression of IFN-γ (Th1), TNF (Th1), IL-2 (Th1), IL-4 (Th2), IL-17a (Th17), CD107a (degranulation), and CD154 (CD40L, activation and B cell help). (B) Data presented in Figure 2E were obtained using a 15-color multi-tetramer panel. Shown is the positive control sample stained with the full panel. Events acquired during steady flow were isolated using a time gate, followed by selection of the live CD3+ events. The markers CD14 and CD19 were used to exclude monocytes and B cells, followed by a singlet gate. CCR7 and MR1 “keeper gates” were used to exclude highly fluorescent events that likely represented artifactual staining. Finally, the lymphocytes were selected based on relative size (FSC) and complexity (SSC). TCR gene usage was examined by antibodies targeting the product of the TCR-α gene TRAV1-2, γδ T cells were identified with a pan-γδ antibody alongside the Vδ2 T cells. The T cells were subdivided based on expression of the CD4 and CD8β co-receptors, and the memory phenotypes were gated separately for each of those subpopulations based upon the expression of CD45RA and CCR7. The gates for each of the tetramers was based upon the relevant fluorescence minus one (FMO) control sample (data not shown). Cells that bound to tetramers nonspecifically, or that bound CD1b regardless of the loaded lipid were identified with the CD1b-Mock tetramer. In an effort to remove fluorophore specific cells from the analysis, the CD1b-Ac2SGL and CD1b-GMM tetramers were included in two channels. These gates were then used to create Boolean subsets for each population of interest. (C) Data presented in Figure 2F were obtained using a 12-color multiparameter flow cytometry panel. The sample shown is a positive control sample. The panel and gating tree are identical to what is shown above, except for the tetramers. This panel includes only the CD1d-α-Galactosylceramide (α-GalCer) tetramer, which is used to identify invariant natural killer T (iNKT) cells. This gate was also drawn using an FMO control.
