# Supplementary material for: IFN-γ independent markers of Mycobacterium tuberculosis exposure among male South African gold miners
Source: eBioMedicine. 2023 Jun 26;93:104678. doi: 10.1016/j.ebiom.2023.104678 (PMC10320233; doi:10.1016/j.ebiom.2023.104678)
Supplement: Supplementary Fig. S2 [file mmc2.pdf]

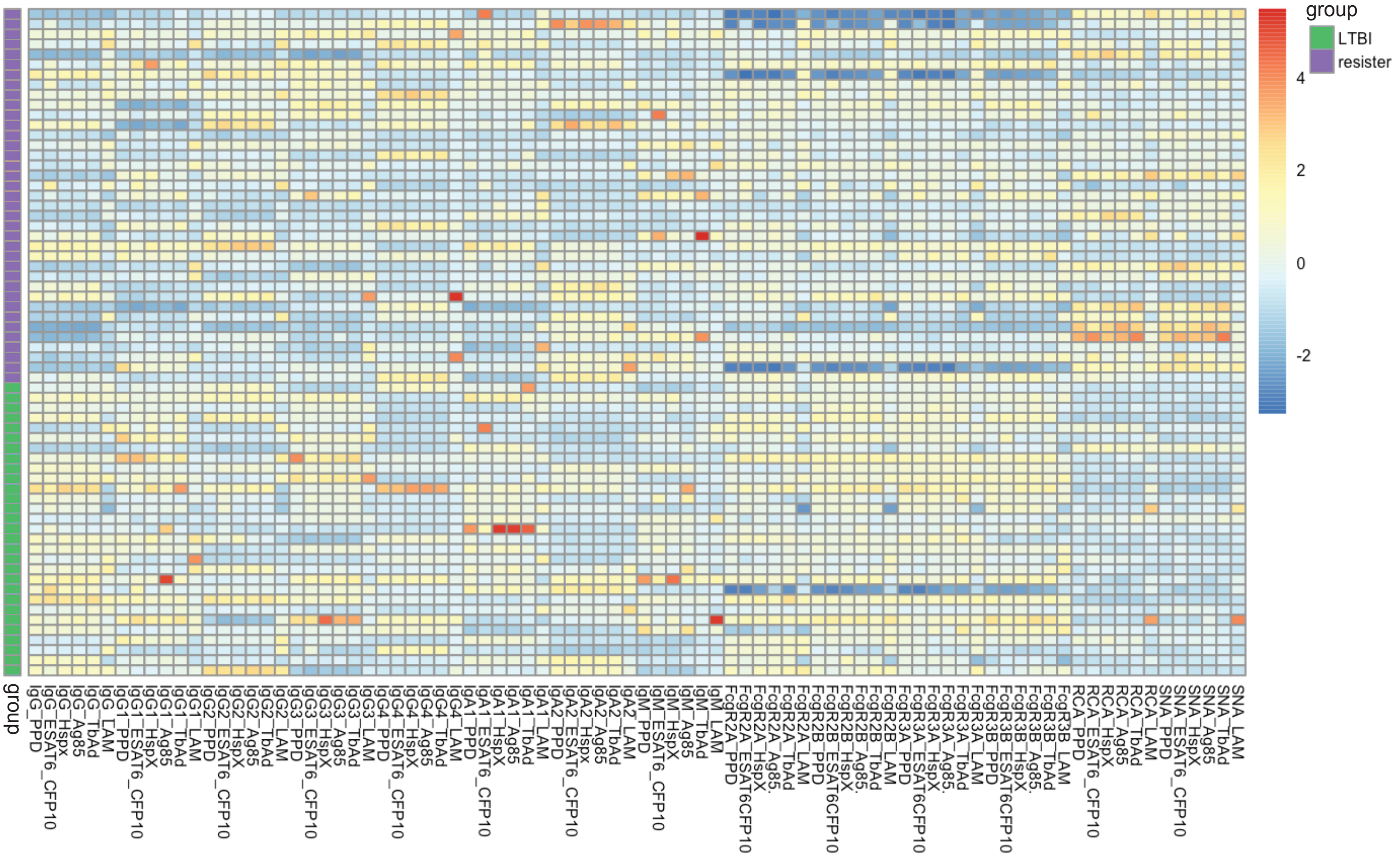

Supplementary Figure 2. Summary heatmap of M.tb-specific antibody data. Luminex was used to measure antibody responses in isotype, subclass, FcR binding, and Fc glycosylation against a panel of M.tb antigens. Heatmap indicates all generated antibody data, Z-scored by assay. Each row represents one individual, and each column represents a single antigen-specific antibody assay.
