# Supplementary material for: IFN-γ independent markers of Mycobacterium tuberculosis exposure among male South African gold miners
Source: eBioMedicine. 2023 Jun 26;93:104678. doi: 10.1016/j.ebiom.2023.104678 (PMC10320233; doi:10.1016/j.ebiom.2023.104678)
Supplement: Supplementary Table S1 [file mmc3.pdf]

		RSTR	LTBI	p value
**T cell profiling**	N	23	23	
**Sex**	Male	23 (100%)	23 (100%)	
**Age, years**	Mean	47.0	49.7	0.23
	SD	6.6	4.1	
**BMI**	Mean	28.5	29.1	0.34
	SD	5.2	3.7	
**Country of birth**	South Africa	17 (73.9%)	18 (78.3%)	0.18
	Lesotho	1 (4.4%)	4 (17.9%)	
	Mozambique	2 (8.7%)	0 (0%)	
	Other	3 (13.0%)	1 (4.4%)	
**Ethnicity**	Black/African	16 (69.6%)	23 (100%)	0.0041
	Other	7 (30.4%)	0 (0%)	
**Working underground**	Yes	18 (78.3%)	15 (65.2%)	0.33
**Years worked underground**	Mean	21.20	24.3	0.16
	SD	6.90	5.50	
**Live in mine housing**	Yes	8 (32.8%)	11 (47.8%)	0.37
**Antibody profiling**	N			
**Sex**	Male	37	30	
**Age, years**	Mean	41 (100%)	30 (100%)	0.43
	SD	47.1	49.0	
**BMI**	Mean	7.0	4.6	0.11
	SD	28.4	30.2	
**Country of birth**	South Africa	4.9	5.5	0.32
	Lesotho	26 (70.3%)	23 (76.7%)	
	Mozambique	2 (5.4%)	4 (13.3%)	
	Other	5 (13.5%)	1 (3.3%)	
**Ethnicity**	Black/African	4 (10.8%)	2 (6.7%)	0.001
	Other	26 (70.3%)	30 (100%)	
**Working underground**	Yes	11 (29.7%)	0 (0%)	0.83
**Years worked underground**	Mean	28 (75.7%)	22 (73.3%)	0.66
	SD	22.6	22.8	
**Live in mine housing**	Yes	6.7	8.0	0.47

Supplementary Table 1. Demographic data for subjects included in T cell and antibody analyses. RSTR and LTBI groups were compared using Mann-Whitney U test (age, BMI, years worked underground) and Chi squared test (sex, country of birth, ethnicity, working underground, and live in mine housing). BMI: body mass index, SD: standard deviation.
