# Supplementary material for: IFN-γ independent markers of Mycobacterium tuberculosis exposure among male South African gold miners
Source: eBioMedicine. 2023 Jun 26;93:104678. doi: 10.1016/j.ebiom.2023.104678 (PMC10320233; doi:10.1016/j.ebiom.2023.104678)
Supplement: Supplementary Table S2–S5 [file mmc4.pdf]

Reagent	Catalog number
Streptavidin-APC	Life Technologies Cat#S868
Streptavidin-ECD	Life Technologies Cat#SA1017
Streptavidin-BV510	BioLegend Cat#405234
Streptavidin-BV650	BioLegend Cat#405232

Supplementary Table 2. Fluorophore-conjugated streptavidin reagents used for generation of tetramers.

Reagent	Catalog number	RRID
LIVE/DEAD Fixable Aqua Dead Cell Stain Kit	Life Technologies Cat# L34966	N/A
CD3 ECD	Beckman Coulter Cat# IM2705U	AB_130860
CD4 APC-A750	Beckman Coulter Cat# A94685	AB_2927448
CD8 PerCP-Cy5.5	BD Biosciences Cat# 341051	AB_400209
IFN-γ V450	BD Biosciences Cat# 560371	AB_1645594
TNF FITC	BD Biosciences Cat# 554512	AB_395443
IL-2 PE	BD Biosciences Cat# 559334	AB_397231
IL-4 APC	BD Biosciences Cat# 554486	AB_398562
CD154 PE-Cy5	BD Biosciences Cat# 555701	AB_396051
IL-17a AF700	BioLegend Cat# 512318	AB_2124868

Supplementary Table 3. Antibody cocktail used for ICS.

Reagent	Catalog number	RRID
CD3 BUV395	BD Biosciences Cat# 563546	AB_2744387
CD4 APC-H7	BD Biosciences Cat# 560837	AB_10563933
CD8β BB700	BD Biosciences Cat# 742229	AB_2740667
CD45RA BUV737	BD Biosciences Cat# 612846	AB_2870168
CD14 BV785	BioLegend Cat# 301840	AB_2563425
CD19 BV785	BioLegend Cat# 363028	AB_2564257
TRAV1-2 BV605	BioLegend Cat# 351720	AB_2563991
Vδ2 AF700	BioLegend Cat# 331416	AB_2687085
Pan-γδ PE-Vio770	Miltenyi Biotec Cat# 130-113-505	AB_2733288

Supplementary Table 4. Antibody cocktail used for combinatorial tetramer panel staining.

Reagent	Catalog number	RRID
IgG PE	Southern BioTech Cat#9040-09	AB_2796601
IgG1 PE	Southern BioTech Cat#9052-09	AB_2796621
IgG2 PE	Southern BioTech Cat#9070-09	AB_2796639
IgG3 PE	Southern BioTech Cat#9210-09	AB_2796701
IgG4 PE	Southern BioTech Cat#9200-09	AB_2796693
IgA1 PE	Southern BioTech Cat#9130-09	AB_2796656
IgA2 PE	Southern BioTech Cat#9140-09	AB_2796664
IgM PE	Southern BioTech Cat#9020-09	AB_2796577
Recombinant human FcγR2A	Duke University Protein Production Core	N/A
Recombinant human FcγR2B	Duke University Protein Production Core	N/A
Recombinant human FcγR3A	Duke University Protein Production Core	N/A
Recombinant human FcγR3B	Duke University Protein Production Core	N/A
Sambuca nigrans agglutinin FITC	VectorLabs Cat#FL-1301	N/A
Ricinus communis agglutinin I FITC	RCA, VectorLabs Cat#FL-1081	N/A

Supplementary Table 5. Secondary detection reagents used for Luminex antibody assays.
